# Filterability of staphylococcal species through membrane filters following application of stressors

**DOI:** 10.1186/1756-0500-3-152

**Published:** 2010-05-30

**Authors:** Laura A Onyango, R Hugh Dunstan, Timothy K Roberts

**Affiliations:** 1 Environmental and Pathogenic Microbiology Laboratory, Faculty of Science and Information Technology, School of Environmental and Life Sciences, Department of Biology, University Drive, Callaghan, 2308, NSW, Australia

## Abstract

**Background:**

Passage of bacterial cells through filter pores has been reported for a number of bacterial species. In this investigation, we tested the filterability of staphylococcal cultures that were exposed to several environmental stress conditions by passing them through 0.22 and 0.45 μm sterile filters, which are industry standards.

**Findings:**

Results showed repeated passage of viable staphylococcal cells through both pore sizes, although more passage was seen through the 0.45 μm pore size. Of the three staphylococcal species, *S. lugdunensis *showed the best passage at relatively higher numbers regardless of the treatment, while both *S. aureus *and *S. epidermidis *showed limited passage or complete inhibition.

**Conclusion:**

The data showed that staphylococcal bacteria were capable of passing through sterile filters in a viable state. There was better passage through 0.45 μm sterile filters than through the 0.22 μm sterile filters. Application of a stress condition did not appear to enhance filterability of these bacterial cultures.

## Background

Microfiltration processes that utilise membrane filters to sterilise liquids have been in use since the early 20^th ^century. They are useful for the removal of contaminants and major pathogens in applications where sterile products are required. Over the years, these filters have been developed further to suit their purpose with pore sizes of 0.2 μm or less, regarded as effective against retention of microbial entities that compromise the sterility of products. Nonetheless, passage of bacterial cells through membrane filters has been repeatedly observed [1-4], even though the mechanisms of cell passage through even the smallest pore sizes is sometimes unknown. Studies conducted on mineral water samples have suggested the presence of bacterial cells as small as 0.15 μm in diameter allowing for the observed passage. Oppenheimer coined these cells ultra-microcells [5]. Although the study of filterable forms was prominent in the early 20^th ^century, interest in this area waned through the years, but has recently been re-ignited with the introduction of the concept of nanobacteria, a class of exceptionally small cells (80-200 nm in diameter) [6]. Although the presence of nanobacteria has been suggested in geology [7] and disease pathology [8,9], their existence and properties are heavily debated concepts, and a recent review even ruled them out as a possible form of life [10,11]. Nonetheless, passage of known bacterial cells through small filter pore sizes is consistently observed and remains a grave concern.

Stressors are known to inhibit cellular processes such as cell size, growth and metabolism [12,13]. *Bacillus subtilis *cells growing under optimal conditions have been described to grow even up to double the cell size of those grown under sub-optimal conditions[13]. Another study [3] showed that older cultures had greater passage through filters than younger cultures. The results of both these studies led us to investigate whether stressed cultures would have smaller cells that would consequently be capable of passing with ease through filter pores. To test this hypothesis, we exposed exponentially growing cultures of *Staphylococcus aureus*, *S. epidermidis*, *S. lugdunensis, Bacillus cereus *and *Escherichia coli *to several stress factors and thereafter tested their filterability through 0.45 μm and 0.22 μm sterile filters, similar to industry standards [14,15]. Our results showed that in most instances, viable staphylococcal cells could repeatedly penetrate through both pore sizes and produce colony growth on horse blood agar (HBA) plates. Growth of these bacterial cultures under stress conditions had a negative impact on filterability since the number of cells passing through the filters was reduced in treatment samples when compared to control samples. However, passage of staphylococcal bacterial cells through the filter pores, when compared to other species, seemed to be favoured by overall shape and/or size.

## Methods

### Bacterial growth

Overnight broth cultures of *S. aureus*, *S. epidermidis*, *S. lugdunensis, B. cereus *and *E. coli *were used to generate fresh cultures which were grown to mid-exponential phase (37°C, 120 rpm) and then harvested. Cell pellets were re-inoculated into fresh broth that was adjusted to a particular stress condition (100 μg/ml of penicillin G and vancomycin in Mueller-Hinton broth, pH 5 in tryptic soy broth, 10% NaCl in nutrient broth). These cultures were allowed to grow for an additional 5 hrs and then filtered. For temperature stress, cultures were grown to mid-exponential phase as described above and thereafter incubated at 4°C for 8 weeks and filtration performed. To test the effect of culture age on filterability, broth cultures grown to mid-exponential phase were kept growing at 37°C without the addition of nutrients and samples taken on a weekly basis and filtered. This was done for a period of 11 weeks.

### Filtration

1 mL aliquot samples (1.0 × 10 ^9 ^cells/mL) were harvested from the stressed cultures above and gently passed through either 0.45 μm or 0.22 μm sterile syringe filters (Durapore^® ^PVDF membrane, Millipore). 100 μl aliquots of the resulting filtrate were then plated onto HBA (Oxoid). These were incubated for 24 h at 37°C and growth recorded as colony forming units/mL (CFU/mL).

### Sub-culture of neat stressed broths

To ascertain the viability of the stressed cultures before filtration, 1:10 serial dilutions were prepared from these cultures and sub-cultured onto HBA plates. These were incubated for 24 h at 37°C and thereafter examined for growth. These plates were also used to determine cell numbers of the original stock solutions.

### Species identification

Colonies growing from filtrate samples were analysed for purity. This was performed by the api Staph^® ^test (bioMérieux) and by PCR of the 16SrRNA gene following the method of Brown *et al*. [16], and the results verified through the NCBI BLAST database http://www.ncbi.nlm.nih.gov/BLAST/.

### Statistical analysis

Experiments were conducted at least three times (each in triplicate) to ensure reproducibility (n = 9). Data was analyzed by ANOVA using Statistica™ (Version 6.1, Statsoft, Tulsa, OK) and represented as mean values ± SD.

## Results

When exponentially growing bacterial cultures were exposed to a stress condition and then filtered, passage through 0.22 and 0.45-μm sterile filter pores was observed for the staphylococci but not for either *E. coli *or *B. cereus *(Table 1). Passage through 0.45-μm filters was greater than passage through 0.22-μm filters for all three staphylococcal species. *S. lugdunensis *showed the greatest passage across all treatments followed by *S. aureus*, while *S. epidermidis *had the least passage. Passage of cells through 0.22-μm sterile filters was also observed for all three staphylococcal species although the numbers were relatively low and even absent in some treatments. Passage of cells through either filter pore did not increase when a stress was applied but in fact decreased under the different stress conditions.

**Table 1:** Results of filtration studies involving *S. aureus, S. epidermidis, S. lugdunensis, E. coli *and *B. cereus *grown in BHI broth and examined under different conditions of growth.

Organism	Growth conditions													
	
	Fresh cultures (10^9^)		Old cultures (11 wks) (10^9^)		4°C (10^9^)		10% NaCl (10^8^)		pH 5 (10^8^)		VA 100 μg ml^-1^ (10^8^)		Pen G 100 μg ml^-1^ (10^8^)	
	
	0.22 μm	0.45 μm	0.22 μm	0.45 μm	0.22 μm	0.45 μm	0.22 μm	0.45 μm	0.22 μm	0.45 μm	0.22 μm	0.45 μm	0.22 μm	0.45 μm
*S. aureus*	1.7 ± 1.9	0.3 ± 0.6	0.1 ± 0.3	12.2 ± 36.3	6 ± 17	4 ± 6	-	38.5 ± 40.8	-	10.5 ± 11.4	-	-	-	-

*S. epidermidis*	-	57.8 ± 53.6	0.1 ± 0.3	5.4 ± 15.2	-	-	-	3.9 ± 8.7	-	5.5 ± 5.5	-	-	-	-

*S. lugdunensis*	0.7 ± 1.4	185.3 ± 46.4	0.4 ± 1.3	101.4 ± 247	23 ± 41	19 ± 40	-	166.3 ± 140.5	-	140.1 ± 112.9	-	23 ± 31.1	-	-

*B. cereus*	-	-	-	-	-	-	-	-	-	-	-	-	-	-

*E. coli*	-	-	-	-	-	-	-	-	-	-	-	-	-	-

-, no passage recorded; VA-vancomycin; PEN G-penicillin G

Results are represented as mean values of replicate experiments ± SD in colony forming units per mL (cfu/mL).

In most cases, plated filtrate samples produced normal colonies on HBA. However, plated filtrates from old cultures sometimes grew as a mixed population of size variants, with small colonies (≤ 1 mm) being the majority in these samples. These colonies were also analysed for species identification alongside the normal colonies by initially performing the API ^® ^Staph test. Although results of the API were sometimes inconclusive for the smaller colonies, PCR-based assays confirmed the identity of both colony types as a pure culture and similar to the original samples.

Non-filtered stressed cultures were also diluted and sub-cultured onto fresh HBA to investigate the viability of the cultures following stress application. Following a 24 hr incubation period at 37°C, colony growth was observed for all treatments. However, these stressed cultures produced colonies that were much smaller in size in comparison to control cultures incubated for the same period. Selected colonies were tested for species identity by use of API ^® ^Staph test and confirmed by PCR.

## Discussion

Passage of bacterial cells through filter sizes has been extensively studied especially in relation to water treatment and pharmaceutical applications, and several explanations given for the observed passage even under the most unlikely circumstances. Oppenheimer and others who have studied filterability of microorganisms from natural mineral water attributed this passage to the possible presence of smaller bacterial cells often referred to as ultra-microcells [5,6]. Grinnell's results showed that culture age affected filterability with older cultures being capable of rapid passage through filter pores than fresher cultures [3]. Amberg concluded from his studies that passage through filters was time-dependent with filterable forms detectable only after the initial 10 min interval of filtration [17]. Wang and colleagues concluded from their filtration studies that the overall shape and flexibility of a bacterium affected its passage through small pore sizes [2].

In this present study, we tested the filterability of bacterial cultures through industrial filter pores following application of a range of stress conditions. We observed passage for *S. aureus*, *S. epidermidis *and *S. lugdunensis *through the filter pores while both *E. coli *and *B. cereus *were inhibited suggesting a species-specific advantage and/or shape advantage. Application of a stress condition did not appear to increase filterability of the cultures, since in most treatment cases, the numbers of cells passing through were fewer in comparison to passage of control samples. There was more passage of bacterial cells through 0.45 μm filters than through the 0.22 μm filters, suggesting a wide range of cell sizes were present in these cultures. Passage of staphylococcal cells through the 0.22 μm filter was mostly inhibited suggesting that only a very small proportion of cells were small enough to pass through. Therefore, this pore size was a better choice for sterilization where staphylococci are involved. Although passage through this pore size was observed for fresh, old and temperature stressed cultures, the occurrences were relatively low. A previous study reported that some 0.2 μm filters consisted of a distribution of pore sizes with some at least as big as 0.5 μm in size [18] and although possible, is highly unlikely the reason for the observed result in our study.

From our results, both overall shape and size of the bacterium seemed to be contributing factors to the observed filterability. *B. cereus *and *E. coli *are both rod-shaped bacteria while the staphylococci are spherical shaped, suggesting that the latter's shape was more suitable to penetrating through small pores. Moreover, S*taphylococci *have a reported size range between 0.9-1.3 μm while the *Bacillus *and *E. coli *are 1.2-10 μm long and 0.5-2.5 μm wide [19]. Their larger dimensions could be plausible reasons why their passage through the filter pores was not observed. Nonetheless, passage of *Bacillus *and *E. coli *through 0.2 μm membranes has been reported, although their passage was also found to be dependent on other factors (time and volume of inoculum) [1] not tested here.

Reports have shown that cultures under stress (eg. lack of nutrient availability) have smaller cells than those growing under optimal conditions [13,20]. With this being true, then it would be expected that other forms of stress would consequently have the same effect on cell-size with smaller cells which would then be capable of passing with greater ease through small pore sizes. Exponentially-growing cultures were exposed to a stressor and incubated for an additional 5 hrs to allow for possible "small forms" to develop. Unfiltered stressed samples when sub-cultured showed a reduction in colony size 24 hrs post-incubation when compared to control samples, indicating that the applied stress had indeed affected cell growth and/or division, which was reflected in colony size. Filtered samples showed an increase in filterability for some *S. aureus *treatment samples compared to control samples of the same but most treatments showed reduced passage of cells. In contrast, both the coagulase negative staphylococci (CNS) *S. epidermidis *and *S. lugdunensis *showed a reduced filterability in all instances when the stress factors were introduced. It is possible that the stressors affected the viability of the cells in the population thereby reducing the proportion of viable cells passing through the filters. This was evident when CFU/mL were significantly fewer for non-filtered stressed samples compared to their corresponding control samples. Thus, even though smaller cells may have existed in the population as suggested by Grinnell [3], their numbers may have been relatively low.

One of the more interesting outcomes of this investigation was the greater filterability of *S. lugdunensis *cultures. This species was better able to pass through at relatively higher numbers, regardless of the treatment condition, while both *S. aureus *and *S. epidermidis *showed limited passage or complete inhibition. This observed ability of *S. lugdunensis *to successfully penetrate both 0.45 μm and 0.22 μm sterile filters and grow normal colonies upon filtrate sub-culture, presents concern not only for applications that use micro-filtration as the sole means of sterilization where *S. lugdunensis *is involved, but physiological consequences in disease pathogenesis with this bacterium. *S. lugdunensis *is commonly isolated from and implicated as the cause of several nosocomial infections [21] and the ability of this opportunistic pathogen to pass through into sterile solutions and remain viable poses potential problems for clinical microbiology. Wainwright [1] suggests that the ability of such bacteria to pass through small holes could pose serious pathological consequences in instances where penetration of membranes, host cells and tissues is possible.

## Conclusion

The assumption that filters with pore sizes ≤ 0.45 μm can retain bacterial populations has repeatedly been disproved with the observation of regular passage of cells through 0.45 μm, 0.22 μm and even 0.1 μm sterile filters. Staphylococcal cells are capable of penetrating through small filter pores, probably favoured by a size and shape advantage, and remain viable in sterile solutions. Although this investigation has shown that 0.22 μm filters were better capable of restraining staphylococcal passage than 0.45 μm filters it would be recommended that pore sizes smaller than this be used to ascertain complete sterility of solutions where filtration is the sole method of sterilization. *S. lugdunensis *results suggested that this species may be better suited to penetrate through filter pores and this finding poses pathological and industrial implications where this species may be involved.

## Competing interests

The authors declare that they have no competing interests.

## Authors' contributions

All authors contributed equally to this manuscript. LO conducted all the experiments under the supervision of HD and TR. All authors contributed to the writing of this manuscript. All authors have read and approved the final manuscript.
